# Perioperative glycemic management in adults presenting for elective cardiac and non-cardiac surgery

**DOI:** 10.1186/s13741-023-00302-6

**Published:** 2023-04-29

**Authors:** Roshni Sreedharan, Sandeep Khanna, Andrew Shaw

**Affiliations:** 1 Department of Intensive Care & Resuscitation, Cleveland Clinic Foundation, Cleveland, OH USA; 2 Department of General Anesthesiology, Cleveland Clinic Foundation, Cleveland, OH USA; 3 Department of Cardiothoracic Anesthesiology, Cleveland Clinic Foundation, Cleveland, OH USA; 4 Department of Outcomes Research, Cleveland Clinic Foundation, Cleveland, OH USA

**Keywords:** Perioperative hyperglycemia, Hypoglycemia, Glucose monitoring

## Abstract

Perioperative dysglycemia is associated with adverse outcomes in both cardiac and non-cardiac surgical patients. Hyperglycemia in the perioperative period is associated with an increased risk of postoperative infections, length of stay, and mortality. Hypoglycemia can induce neuronal damage, leading to significant cognitive deficits, as well as death. This review endeavors to summarize existing literature on perioperative dysglycemia and provides updates on pharmacotherapy and management of perioperative hyperglycemia and hypoglycemia in surgical patients.

## Background

Perioperative dysglycemia is associated with adverse outcomes, including an increased risk of postoperative infections, length of stay and mortality in both cardiac and non-cardiac surgical patients (Estrada et al. 2003; Frisch et al. 2010). Although not clearly established, the increased risk posed by hyperglycemia is likely due to the impairment of chemotaxis and phagocytosis, increased expression of adhesion molecules, impaired complement function and nitric oxide generation. This in turn leads to increased inflammation, susceptibility to infection and multi organ dysfunction (Duggan et al. 2017; Lipshutz Angela et al. 2009; Turina et al. 2005). Intensive insulin therapy (IIT) which aims at achieving normoglycemia in hospitalized and critically ill patients was expected to reverse these risks. Although the Leuven study published in 2001 demonstrated robust results with a significant reduction in mortality and morbidity in surgical intensive care patients on IIT (Berghe et al. 2001), studies which followed were unable to replicate these results. In fact, they demonstrated an increased risk of severe hypoglycemia, mortality and morbidity in patients who received IIT (Finfer et al. 2009; Gandhi et al. 2007; Brunkhorst et al. 2008). Although hyperglycemia is associated with adverse outcomes, achieving normoglycemia in all perioperative patients may not be the answer. The data we have so far favors a more moderate approach to glycemic management in the perioperative period, minimizing hypoglycemia and perhaps individualizing therapy based on the patients preexisting glycemic status.

This review examines the prevalence of dysglycemia and provides an update on the management of cardiac and non-cardiac surgical patients with preexisting diabetes, hyperglycemia, and hypoglycemia in the perioperative period. Perioperative management of the parturient with diabetes is beyond the scope of this manuscript and has not been addressed in it.

## Diabetes, hyperglycemia, and the burden of undiagnosed diabetes

In 2014, 8.5% of the world’s adult population was afflicted with diabetes. 1.5 million deaths in 2019 were directly attributed to Diabetes (Organisation WH 2022). According to the 2020 National diabetes Statistics report, 10.5% of the US population is afflicted with diabetes. Of these, approximately 21.4% are undiagnosed (Control CfD 2020). Patients with diabetes are more likely to undergo surgery as compared to those without (Smiley and Umpierrez 2006). Diabetes confers a high risk of coronary artery disease, which is optimally treated with Coronary Artery Bypass Grafting (CABG) when multiple vessels are involved (Naito and Miyauchi 2017). The micro and macrovascular changes, multiorgan dysfunction and obesity often seen in patients with diabetes leads to them presenting for orthopedic and other surgical procedures. Furthermore, when they do have surgical procedures, these patients carry a higher perioperative risk of mortality and morbidity (Frisch et al. 2010). Undiagnosed diabetes is prevalent worldwide. Undiagnosed diabetes is a state wherein the patient has diabetes (fasting blood glucose ≥ 126 mg/dl or HbA1C ≥ 6.5%) but has not yet been diagnosed (Levy and Dhatariya 2019). In 2021, according to the International Diabetes Federation (IDF) data, almost one in two adults with diabetes were unaware of their diabetic status (44.7%) (Ogurtsova et al. 2022). As expected, the burden of both diagnosed and undiagnosed diabetes is significant in the cardiac and non-cardiac surgical population. A large prospective observational study evaluating the impact of diabetes on adverse outcomes after surgery, found that about 27% (2047/7565) of the patients had diabetes and 3% (236/7565) had undiagnosed diabetes (Yong et al. 2018). In the cardiac surgical population, a retrospective study looking at 7310 patients documented a prevalence of 29.6% for diagnosed diabetes and 5.2% for undiagnosed diabetes (Lauruschkat et al. 2005). In the non-cardiac surgical population, Abdelmalak and colleagues observed that 10% (3426/33,923) of the patients in their cohort had undiagnosed diabetes (Abdelmalak et al. 2010). A more recent large retrospective cohort study looking at undiagnosed diabetes and perioperative outcomes, observed that patients with undiagnosed diabetes have a significantly increased risk of one-year mortality compared to patients without diabetes (Teo et al. 2020). Cardiac surgical patients with undiagnosed diabetes seemed to carry a higher likelihood of reintubation, prolonged mechanical ventilation and mortality when compared to their diagnosed diabetic counterparts (Lauruschkat et al. 2005). These outcomes are perhaps related to lack of recognition, treatment, and preoperative risk stratification in these patients (Sreedharan and Abdelmalak 2018). It is recognized that some of the patients who present with perioperative hyperglycemia and not diagnosed diabetes could be in a state of “stress hyperglycemia”. Longitudinal studies have shown that a large proportion of these patients with new hyperglycemia have confirmed diabetes at 1 year (Mazurek et al. 2010). Hemoglobin A1C (HbA1C) is a useful tool to help differentiate stress hyperglycemia from undiagnosed diabetes (Mazurek et al. 2010). In patients with hyperglycemia, a HbA1C greater than 6.5% helps establish a diagnosis of Diabetes (Umpierrez et al. 2002). Furthermore, ascertaining their chronic glycemic state might be valuable in determining their glycemic goals for the perioperative period (Sreedharan and Abdelmalak 2018; Abdelmalak and Lansang 2013).

### The stress of surgery and anesthesia

**Fig. 1:**
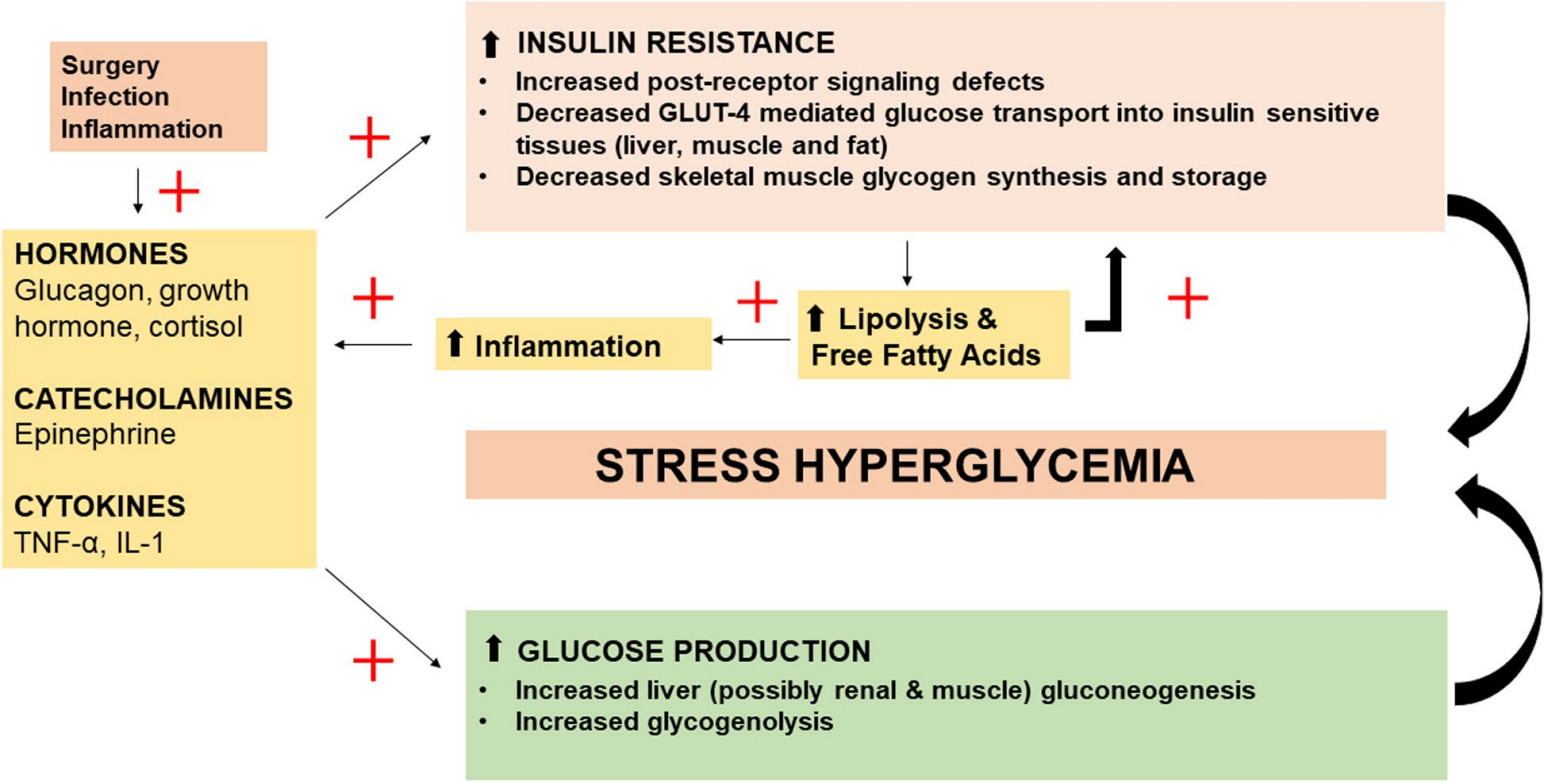
Genesis of stress hyperglycemia. Legend: GLUT-4: Glucose transporter 4, IL-1: Interleukin-1, TNF: Tumor necrosis factor

A complex interplay between counter regulatory hormones (catecholamines, glucagon, glucocorticoids, and growth hormone), circulating cytokines and peripheral insulin resistance plays a role in the genesis of stress hyperglycemia (see Fig. 1) (Dungan et al. 2009; Sreedharan and Abdelmalak 2017).

Due to post-receptor insulin signaling defects, there is insulin resistance and an increase in non-insulin mediated glucose uptake. Unregulated gluconeogenesis and glycogenolysis results in hyperglycemia, oxidative stress, and glucotoxicity (Dungan et al. 2009). Furthermore, insulin resistance increases lipolysis, releasing free fatty acids. Free fatty acids promote inflammation and create an environment of lipotoxicity along with the glucotoxicity. The hyperglycemia that occurs during periods of stress is likely a normal adaptive response to increase chances of survival (Sreedharan and Abdelmalak 2017; Marik and Bellomo 2013). The expectation is that this hyperglycemia would resolve spontaneously once the stress dissipates. Extrinsic administration of glucocorticoids used to prevent postoperative nausea and vomiting (PONV), attenuate inflammatory response, and improve lung function tends to further accentuate this process (Abdelmalak et al. 2013; Morariu et al. 2005; Chaney et al. 2001). The magnitude of stress hyperglycemia in the perioperative period tends to depend on the extent of surgery and type of anesthesia (Clarke 1970). Intraabdominal and intrathoracic procedures and utilization of general anesthesia tend to be associated with a more profound and persistent hyperglycemia (Clarke 1970; Hu and Mohammed 2003). Although adaptive, the adverse effects of uncontrolled hyperglycemia have been well established. In fact, studies have recognized that in cohorts of both ICU and non-ICU patients in the hospital, patients with new onset hyperglycemia had a higher mortality and morbidity as compared to their diabetic counterparts (Umpierrez et al. 2002; Dungan et al. 2009). Large randomized controlled trials have shown that attempts to completely correct this response have proven to be detrimental (Finfer et al. 2009). Currently, the American Diabetes Association (ADA) and major surgical societies endorse a moderate approach to glycemic control, limiting the risk of hypoglycemia (Association AD 2021).

## Diabetes mellitus and hyperglycemia—definition and recognition

During the perioperative period when a patient presents with hyperglycemia, it is imperative to recognize if the patient has diabetes, stress hyperglycemia or undiagnosed diabetes to provide appropriate treatment and follow-up.

According to the American Diabetes Association a diagnosis of Diabetes is made if any one of the following criteria are met (Association 2020).

- Fasting plasma glucose greater than 126 mg/dL (7 mmol/L)
- Postprandial plasma glucose > 200 mg/dL (11.1 mmol/L after 2 h of a 75-g oral glucose load
- HbA1C > 6.5%
- Classic symptoms of hyperglycemic crisis with a random glucose > 200 mg/dL (11.1 mmol/L)

The ADA recommends that a HbA1C be tested in any patient in the hospital, with a blood sugar > 140 mg/dl (7.77 mmol/L) if there was not one performed in the past 3 months. This can help diagnose diabetes and differentiate it from stress hyperglycemia (Association AD 2021).

## Glucose monitoring in the perioperative period

Point of care (POC) glucometers using capillary blood are often used to monitor and manage blood glucose. Although convenient, it is important to recognize the limitations associated with them. Variables including rapidly changing hematocrit, hypoxia, acidosis, use of vasopressors and peripheral edema can affect their accuracy. As per the guidelines put forth by the Food and Drug Administration, 99% of POC readings greater than 70 mg/dl should be within 10% of central laboratory reference values and all blood glucose readings less than 70 mg/dl be within 7 mg/dl. Glucometers in several hospitals may not meet these required standards (Duggan et al. 2017). Overall, it has been recognized that they are not accurate for the intensive monitoring and treatment of critically ill patients (Critchell et al. 2007; Hoedemaekers et al. 2008; Kanji et al. 2005; Finfer et al. 2013). The other options for monitoring blood glucose in the perioperative period include central laboratory testing and blood gas analysis. Central laboratory testing of whole blood glucose is the gold standard but turnaround time for the results limit its practical utility when testing is required every 1–2 h. Arterial blood gas sample analysis has widely been used for monitoring blood glucose. It is close to the laboratory standard and feasible to perform with quick reports of critical results (Kanji et al. 2005). Blood glucose measurement using arterial samples and a blood gas analyzer are noted to be associated with fewer errors when compared to capillary samples or analysis of arterial samples using a glucometer (Herpe and Mesotten 2012).

Continuous glucose monitoring (CGM) technology has gained increasing popularity in diabetic patients with its ability to detect episodes of dysglycemia early and consequently maintain blood glucose within target range. CGM sensors could be invasive (intravascular), minimally invasive (subcutaneous) or non-invasive (transdermal) (Galindo et al. 2020). Although two invasive CGM’s have received FDA clearance for in-hospital use, the most commonly utilized CGMs are subcutaneous wherein the sensor is placed in the subcutaneous space to measure the glucose concentration in the interstitial fluid, which is then transmitted every few minutes to a receiver carried by the patient. Although intraoperative volume shifts, edema, and vasopressor requirements leading to interstitial hypoperfusion and subsequent measurement inaccuracies have deterred the perioperative use of CGM, they have demonstrated accuracy and reliability in ICU patients in shock and on vasopressors (Holzinger et al. 2009; Partridge et al. 2016). A recent study looked at the frequency and duration of perioperative dysglycemia using CGM. The results were astounding. Hypoglycemic events lasting more than 15 min, occurred in 43% of all patients and 70% of patients with type 1 diabetes. Particularly, diabetic patients seemed to be more prone to both hypo- and hyperglycemic episodes in the perioperative period (Carlsson et al. 2021). Compared to POC glucose testing, real-time CGM has been noticed to recognize early and hence decrease both hypoglycemia and hyperglycemia episodes in hospitalized patients (Singh et al. 2020; Fortmann et al. 2020). The most recent guidelines published by Center for Perioperative Care (CPOC) does not recommend the utilization of CGM in sedated or anesthetized surgical patients in procedures lasting longer than 60 min (CPOC 2022). Although not yet widely used, CGM might have a role in the perioperative management of both diabetic and non-diabetic patients in the future. The impact of appropriate early detection and intervention for dysglycemic episodes, and reduction of glycemic variability can be profound. Large-scale studies to validate the accuracy of CGM in the perioperative period would be valuable in lending more evidence to its value.

## Preoperative evaluation of patients with diabetes

Optimal preoperative evaluation and preparation of patients with diabetes involves a detailed investigation for the presence and impact of macro and microvascular complications of diabetes, chronic glycemic control and antihyperglycemic therapy.

**Fig. 2:**
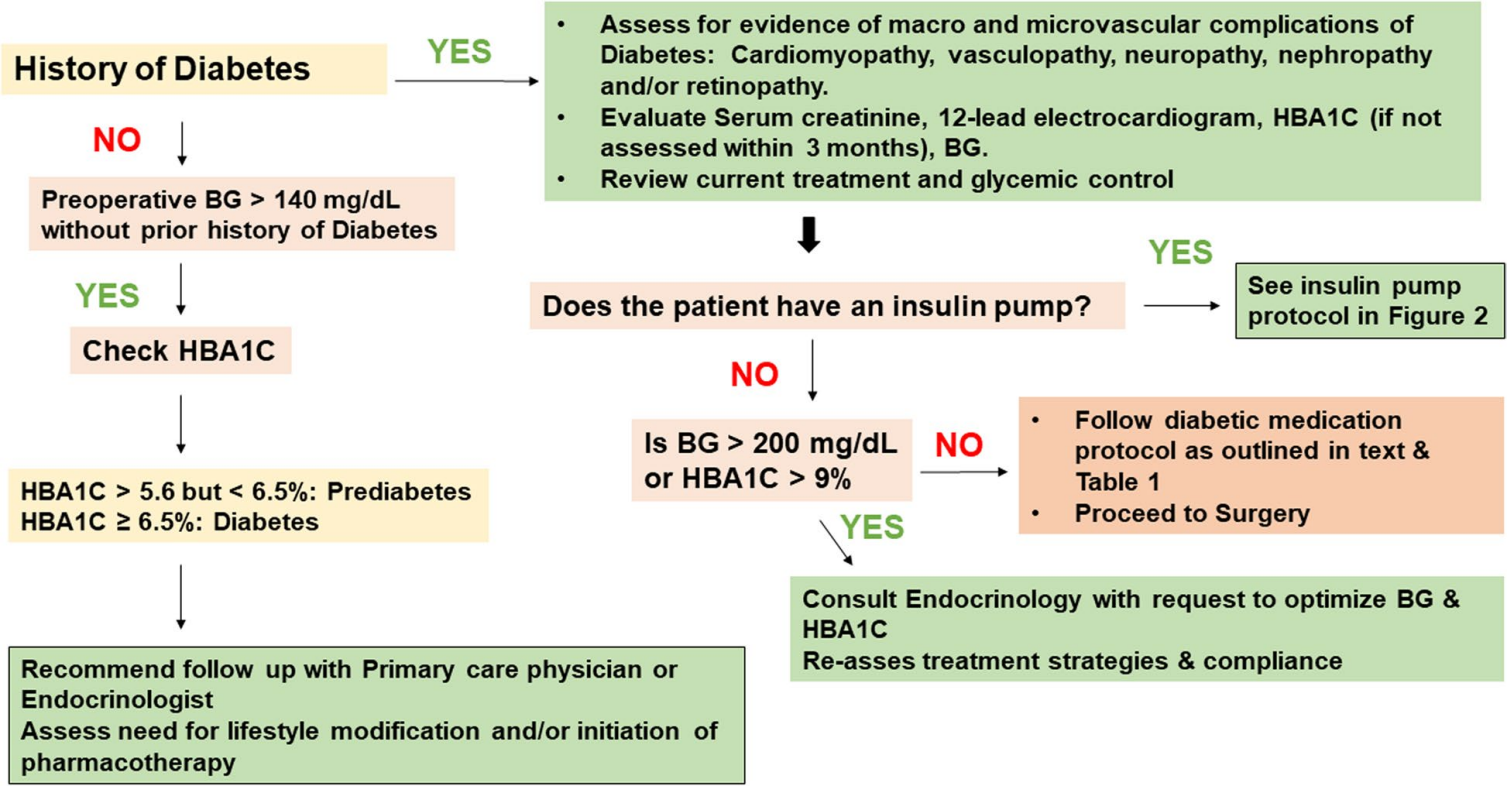
Preoperative management algorithm for patients with diabetes. Legend: BG (blood glucose), DM (diabetes mellitus), HBA1C (glycated hemoglobin)

An approach to the preoperative evaluation of a patient with diabetes is outlined in Fig. 2.

### Preoperative management of oral antihyperglycemic therapy

A large proportion of adults with type 2 diabetes are on oral hypoglycemic agents (OHA) for glycemic management. The predominant concern with these agents is the potential for hypoglycemia and this risk seems to be higher with agents like sulfonylureas and glinides that increase or stimulate insulin secretion (Sreedharan and Abdelmalak 2018). It is recommended that these agents be discontinued on the day of surgery due to the risk of hypoglycemia (Umpierrez et al. 2012). The ADA recommends metformin as the preferred initial pharmacological agent of choice for the treatment of type 2 diabetes (Association AD 2021). The Society of Ambulatory Anesthesia (SAMBA) consensus statement on perioperative blood glucose management recommends the continuation of metformin the day before surgery and resuming therapy on the day of surgery once a regular diet is resumed (Joshi et al. 2010). Ifutilization of contrast dye is anticipated, metformin is discontinued at the onset of the preoperative fast and restarted postoperatively once a normal diet is resumed. The most recent guidelines for perioperative care for patients with diabetes, published by the CPOC recommends continuing metformin on the day of surgery (once or twice daily dose) if renal function is normal and no contrast use is anticipated. If taken thrice daily, the lunchtime dose is omitted (CPOC 2022). In the event of perioperative renal dysfunction, metformin is discontinued and restarted once the renal function normalizes (Duggan et al. 2017). The American College of Endocrinology (ACE) and American College of Clinical Endocrinologists (ACCE) recommend holding sodium co transporter 2 inhibitors, 24 h before elective surgery or invasive procedures due to the risk of Euglycemic Diabetic Ketoacidosis (Handelsman et al. 2016). Recent studies have attested to the safety of Dipeptidyl peptidase-4 (DPP-4) inhibitors in surgical patients (Umpierrez et al. 2013). The current recommendation is to continue these agents on the day of surgery and through the perioperative period (Duggan et al. 2017).

### Preoperative management of insulin therapy

All patients with type 1 and some patients with type 2 diabetes take insulin as a part of their regimen for glycemic control. Due to the absolute deficiency of insulin in patients with type 1 diabetes, their insulin regimen covers the basal requirement as well as anticipated nutritional and unanticipated accidental hyperglycemia. Even during fasting, these patients need supplemental insulin to prevent ketosis (Sreedharan and Abdelmalak 2018). The recommendation is to administer 80% of their basal insulin dose the evening before and morning of surgery (Mendez and Umpierrez 2013). Although long-acting insulin analogues like glargine and detemir do not peak and are unlikely to lead to hypoglycemia in the perioperative fasting state, to be safe and minimize the risk of hypoglycemia, the recommendation is to reduce the dose by 20–30% the night before and the day of surgery, if on a twice daily dosing (Barker et al. 2015; Vann 2014; Demma et al. 2017). We recommend a 25% reduction in the dosing the night before and 50% reduction in the dose on the day of surgery for long-acting insulin analogues. The risk of hypoglycemia is higher with intermediate acting and premixed insulins. A 25–50% reduction in the dose is recommended the day before and day of surgery (Joshi et al. 2010; Barker et al. 2015). On the day of surgery, if the blood glucose is greater than 200 mg/dl (11.11 mmol/L), 50% of the dose is administered. If the blood glucose is less than 200 mg/dl, the dose is omitted (Sreedharan and Abdelmalak 2018).

Table 1 outlines the various antihyperglycemic agents, their physiological effects, and preoperative management.

**Table 1:** Oral and injectable antihyperglycemic agents—preoperative management

Class	Drug	Physiological effect	Risk of hypoglycemia	Day before surgery	Day of surgery
Biguanides	Metformin	Decrease hepatic glucose production	Low	Continue regular use	Omit dose if eGFR < 60 ml/min/1.73 m^2^ or if IV contrast anticipated. If not, take morning dose
Sulfonylureas	Gliclazide, glipizide, glimepiride	Increase insulin secretion	Moderate to high	Continue regular use	Omit dose
Thiazolidinediones	Rosiglitazone, pioglitazone	Increase insulin sensitivity	Low	Continue regular use	Omit dose
Glinides	Nateglinide, repaglinide	Increase insulin secretion	Moderate	Continue regular use	Omit dose
Alpha glucosidase inhibitors	Acarbose	Slow intestinal carbohydrate absorption	Low	Continue regular use	Omit dose
Dipeptidyl peptidase-4 inhibitors	Sitagliptin, saxagliptin	Glucose-dependent increase in insulin section and decrease in glucagon secretion	Low	Continue regular use	Continue regular use
Glucagon like peptide-1 analogs	Exenatide, liraglutide	Glucose-dependent increase in insulin secretion	Low	Continue regular use	Omit dose
Sodium glucose co transporter-2 inhibitors	Dapaglifozina, canagliflozin	Decreases glucose reabsorption by the kidney	Low	Omit dose	Omit dose
Long acting basal insulin	Lavemir, Lantus		Low	Take 75% of dose	Take 50% of dose
Mixed insulin; combination of long and short acting, i.e., 70/30 or 75/25			Moderate to high	Take 75% of dose	If morning blood glucose is a. > 200 mg/dL take 50% of dose, b. ≤ 200 mg/dL omit dose

### Perioperative management of insulin pumps

An insulin pump delivers a continuous basal infusion and bolus doses of insulin as programmed. From being used exclusively in type 1 diabetes, there is an increase in utilization of insulin pumps for the treatment of type 2 diabetes. There has been extensive research and development in this area from first generation insulin pumps to the second-generation fully automated insulin closed loop pumps. Closed loop pumps are an advancement wherein the insulin pump is paired with a CGM, which enables alteration of the insulin infusion based on the glucose level and a specific algorithm. There are few hybrid-closed loop systems that are commercially available and few still in development (Templer 2022). When patients with an insulin pump present for their preoperative evaluation it is important to evaluate and document the type of device, the site of infusion, type of pump, formulation, and dosing regimen. An endocrinology consult is obtained preoperatively if it is an elective procedure, and the patient is likely to be admitted to the hospital postoperatively.

One of the challenges and controversies surrounding the perioperative use of insulin pumps is their incompatibility with diathermy per manufacturer recommendations. CPOC recommends multidisciplinary discussion and shared decision making in this situation reviewing the risks, benefits and alternatives available (CPOC 2022). Their most updated guideline outlines the pre-requisites for the safe perioperative use of continuous subcutaneous insulin infusions including optimal preoperative glycemic control, ability to keep pump away from the surgical site, ability to position the earthing plate and diathermy appropriately away from the pump, and the ability to initiate a continuous infusion if needed (CPOC 2022). Figure 2 outlines the protocol developed with multidisciplinary collaboration at the Cleveland Clinic, for the perioperative glycemic management in patients with insulin pumps. The basal dose of insulin through the pump is maintained during the preoperative fasting period. For short procedures, the insulin pump could be maintained with the basal infusion on for the intraoperative period. In that circumstance, blood glucose should be monitored every hour. If the procedure is emergent or is anticipated to be longer than 3 h, the insulin pump is disconnected, and an intravenous insulin infusion is utilized to maintain a target blood glucose of 140–180 mg/dl (7.7–10 mmol/L) (Partridge et al. 2016; Abdelmalak et al. 2012).

**Fig. 3:**
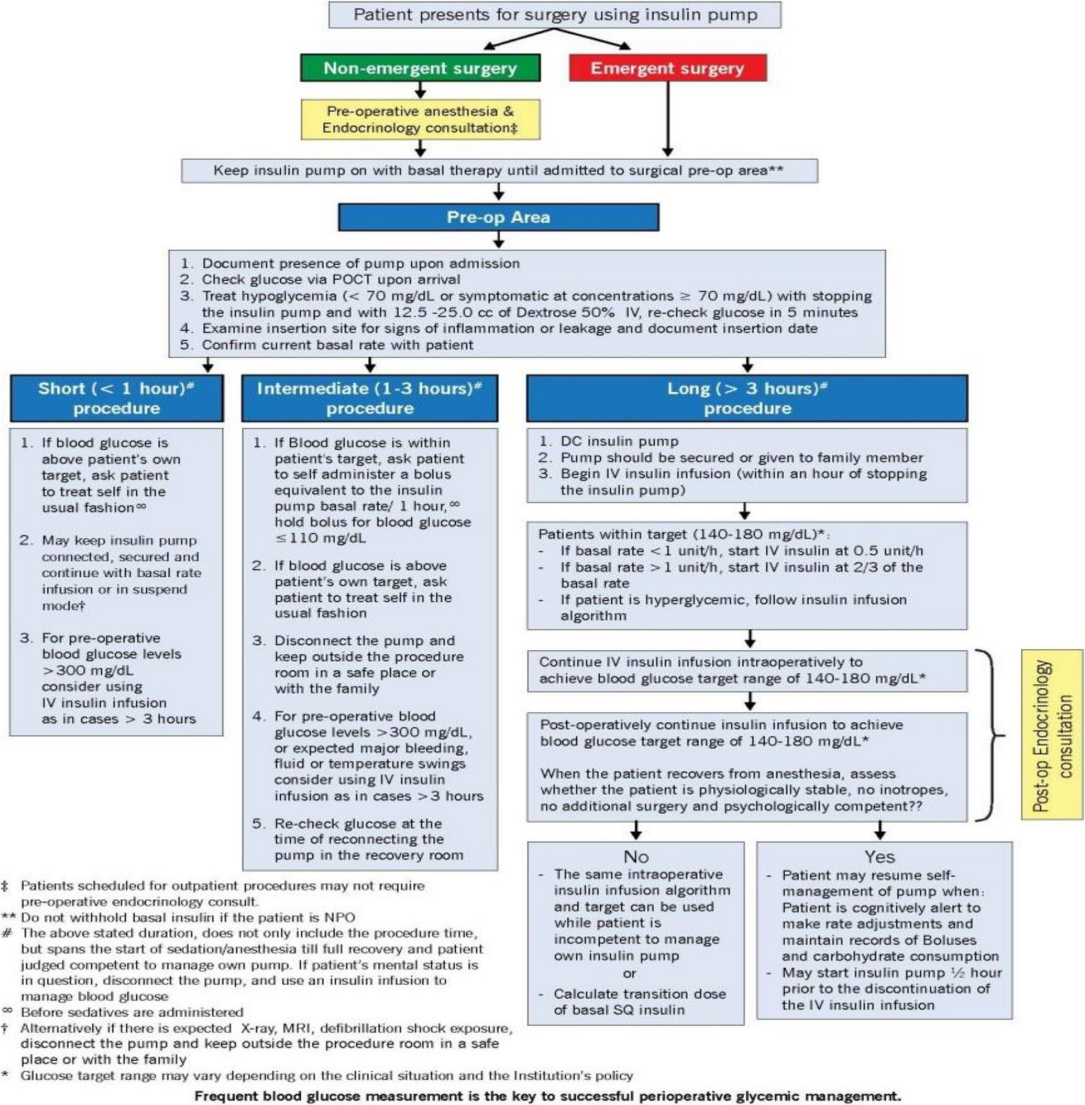
Perioperative glycemic management in insulin pump patients undergoing non-cardiac surgery. Legend: Courtesy Cleveland Clinic center for art and photography, Cleveland Clinic

Figure 3 outlines the perioperative management of a patient on an insulin pump.

## Perioperative glycemic goals in non-cardiac and cardiac surgical patients

Based on the most recent evidence, the consensus is in favor of a moderate goal for glycemic control in perioperative patients with or without diabetes.

### Non-cardiac surgery

Consensus guidelines put forth by the SAMBA, The AACE task force and ADA, and Society of Critical Care Medicine (SCCM) recommend maintenance of target blood glucose less than 180 mg/dl (Joshi et al. 2010; Moghissi et al. 2009; Jacobi et al. 2012). In patients with or without diabetes in both surgical and medical ICU’s, The American College of Physicians (ACP) advocates against the use of intensive insulin therapy (Qaseem et al. 2011). Insulin therapy is initiated for persistent hyperglycemia over 180 mg/dl (10 mmol/L). Once initiated, insulin infusion is titrated to achieve a target blood glucose between 140 and 180 mg/dl (7.7–10 mmol/L) (Association AD 2021).

During the perioperative period, a glycemic target tighter than 140 mg/dl (7.7 mmol/L) has not shown to confer an advantage over a target less than 180 mg/dl (10 mmol/L) (Sathya et al. 2013).

### Cardiac surgery

The report from the Society of Thoracic Surgeons’ (STS) workforce on evidence-based surgery put forth their guidelines on blood glucose management during adult cardiac surgery. They recommend maintaining serum glucose less than or equal to 180 mg/dl (10 mmol/L) for 24 h after cardiac surgery (Lazar et al. 2009). Once initiated, the infusion is titrated to maintain a goal of 140–180 mg/dl (Duggan et al. 2017). A tighter target (100–140 mg/dl) (5.5–7.7 mmol/L) has not shown to improve perioperative outcomes in this subset of patients as well (Umpierrez et al. 2015; Lazar et al. 2011; Desai et al. 2012).

In both cardiac and non-cardiac surgical patients, intensive insulin regimens aimed to achieve tighter blood glucose targets have found to not be beneficial and confer a high risk of hypoglycemia and increased glucose variability and hence not recommended.

### Glycemic domains and variability

The three domains glycemic control include hyperglycemia, hypoglycemia, and glycemic variability. Each of these domains have shown to be independently associated with increased mortality in critically ill patients (Krinsley 2003; Krinsley 2008; Krinsley et al. 2011). A large study evaluating these three domains and their impact on glycemic management in the ICU revealed an association of hyperglycemia and glycemic variability with increased mortality in patients without diabetes. Hypoglycemia on the other hand was associated with mortality in both groups (Krinsley et al. 2013). Poor preadmission glycemic control increased the risk of mortality when patients experienced a lower than usual blood sugar or “relative hypoglycemia” in the ICU. Maintenance of blood glucose closer to the normal range seemed to improve survival in patients with good preadmission glycemic control (Krinsley et al. 2012). A retrospective analysis of 4302 cardiac surgical patients evaluating the effect of glycemic variability on adverse outcomes observed that increased postoperative glycemic variability was associated with an increased risk of mortality and overall morbidity (Duncan et al. 2010). The results of a prospective single center observational cohort study evaluating the effect of increased glycemic variability on predicting outcomes after CABG surgery suggest that postoperative glycemic variability is a predictor of Major Adverse Events (MAEs), particularly deep sternal wound infection. Of note, they noticed that postoperative glycemic variability was increased in patients with poor preoperative glycemic control (Subramaniam et al. 2014). Appropriate preoperative optimization of glycemic status and prevention of wide swings in blood glucose are of paramount importance in optimizing postoperative outcomes. Notably, there has been an increasing recognition that one glycemic target might not fit all, emphasizing the need to individualize therapy based on the patient’s preexisting glycemic status (Krinsley 2013).

### Intraoperative management—cardiac surgery and non-cardiac surgery

Insulin therapy is initiated for intraoperative blood glucose levels higher than 180 mg/dl (10 mmol/L). Either subcutaneous or intravenous insulin therapy could be utilized to achieve the glycemic goal depending on the duration and complexity of the surgical procedure as well as the time to resumption of oral intake. Subcutaneous correctional insulin therapy with blood glucose monitoring every 2 h can be considered for intraoperative glycemic management in patients undergoing ambulatory surgeries, procedures shorter than 4 h in duration, and those in which hemodynamic stability and early resumption of oral intake are anticipated (Joshi et al. 2010). When subcutaneous insulin is utilized to treat intraoperative hyperglycemia, particular attention needs to be paid to factors that increase insulin sensitivity and resistance, to ensure that insulin is dosed appropriately, to prevent swings of hypo and hyperglycemia (Duggan et al. 2017).

An insulin infusion with hourly blood glucose monitoring is warranted for glycemic control in cardiac surgical patients and patients undergoing any procedure wherein hemodynamic fluctuations, fluid shifts, use of vasopressors, temperature changes, or lengthy operative times (> 4) are anticipated (Duggan et al. 2017).

## Postoperative glycemic management—cardiac and non-cardiac surgery

### Hyperglycemia

The goal is to initiate insulin therapy if blood glucose levels are over 180 mg/dl and maintain between 140 and 180 mg/dl (7.7–10 mmol/L) in both postoperative cardiac and non-cardiac surgical patients to optimize outcomes (Duggan et al. 2017). As per the STS guidelines, in cardiac surgical patients, blood glucose less than 180 mg/dl (10 mmol/L) is maintained for at least 24 h after surgery. If patients are critically ill or in the intensive care unit, an insulin infusion with blood glucose measurements every hour is utilized to achieve this goal to ensure appropriate dosage, titration, and management. In patients who are otherwise well and in the Post-Anesthesia Care Unit (PACU), correctional subcutaneous insulin with blood glucose checks every 2 h can be utilized to achieve a target of 140–180 mg/dl (7.7–10 mmol/L). When transitioning from the PACU to the regular nursing floor, correctional insulin alone may be insufficient in diabetic patients for appropriate glycemic control. Long-acting basal insulin with or without prandial insulin is recommended to attain the goal and minimize variability (Umpierrez et al. 2013; Umpierrez et al. 2011). Depending on the nutritional status of the patient, either a “basal bolus” or “basal plus” insulin regimen could be utilized to achieve the glycemic goal. Utilization of a long-acting basal insulin with prandial rapid acting insulin is referred to as the “basal bolus” approach. Better blood glucose control in comparison to sliding scale insulin and a reduction in the incidence of postoperative infections were seen in general surgical patients with this approach (Umpierrez et al. 2011). Utilization of a long-acting basal insulin with correctional rapid acting insulin is referred to as the “basal plus” approach. Improved glycemic control has been seen in general surgical patients with this approach as compared to sliding scale alone if their caloric intake was poor caloric intake (Umpierrez et al. 2013). Overall, in surgical patients, the “basal bolus” approach is favored when nutritional intake is adequate, and the “basal plus” approach is favored when their nutritional intake is limited (Duggan et al. 2017).

All patients who continue to need insulin infusion to maintain their glycemic goals in the ICU need to be transitioned to a scheduled subcutaneous regimen to prevent rebound hyperglycemia when ready to be transferred to a regular nursing floor (Schmeltz et al. 2006). The subcutaneous dose of insulin to be given is calculated from the total daily dose (TDD) of insulin utilized by the patient based on the infusion. Seventy percent of this dose is administered as a basal insulin. The remaining 30% is administered as a prandial dose once the dietary intake is adequate (Smiley and Umpierrez 2010). The basal dose is administered 2 h before the insulin infusion is stopped to prevent rebound hyperglycemia. Patients who do not carry a diagnosis of diabetes and who require a low-dose insulin infusion to maintain their glycemic target might not need a basal insulin dose but might require correctional subcutaneous insulin when transitioning to the regular nursing floor (Duggan et al. 2017; Umpierrez et al. 2015).

### Hypoglycemia

Hypoglycemia is the most common adverse event associated with insulin therapy (Duggan et al. 2017). The National Diabetes Inpatient Audit (NaDIA) 2020 report published in 2021, identifies hypoglycemic rescue as the number one adverse event (National Diabetes Inpatient Audit 2021).It is independently associated with increased mortality and morbidity (Finfer et al. 2009). Severe hypoglycemia, albeit for a short duration, can induce neuronal damage, especially in the vulnerable parts of the cortex, hippocampus, and basal ganglia. This could translate into significant deficits with cognition, memory, and orientation in patients (Jackson et al. 2018; Languren et al. 2013). Sedation and general anesthesia mask the symptoms of hypoglycemia. Heightened vigilance and consistent monitoring protocols are vital to the recognition of hypoglycemia in the perioperative period.

### Definition of hypoglycemia

Hypoglycemia can be defined in five categories (Seaquist et al. 2013; Cryer 2005)

- Severe hypoglycemia—symptoms requiring assistance from another individual, neuroglycopenic symptoms, seizures or coma, and reversal of symptoms with the administration of glucose, which is diagnostic.
- Documented symptomatic hypoglycemia—measured plasma glucose < 70 mg/dl (3.8 mmol/L)
- Asymptomatic hypoglycemia—measured plasma glucose < 70 mg/dl (3.8 mmol/L) without symptoms of hypoglycemia.
- Probable symptomatic hypoglycemia—measured plasma glucose not available but symptoms presumed to be due to a plasma glucose < 70 mg/dl (3.8 mmol/L)
- Pseudo hypoglycemia or relative hypoglycemia—symptoms of hypoglycemia in a person with diabetes but with a measured plasma glucose > 70 mg/dl (3.8 mmol/L)

The severity of hypoglycemia is defined by levels recognized by the ADA (Association 2020)

Level 1—Blood glucose concentration < 70 mg/dl (3.8 mmol/L) but >= 54 mg/dl (3 mmol/L)

Level 2—Blood glucose concentration < 54 mg/dl (3 mmol/L)

Level 3—Severe event. Altered mental or physical functioning requiring assistance from another individual

Independent of clinical symptoms of hypoglycemia, level 1, or a blood glucose < 70 mg/dl (3.8 mmol/L) is considered clinically significant. Neuroglycopenic symptoms tend to manifest when blood glucose is less than 54 mg/dl (3 mmol/L) (Association 2020).

### Recognition of hypoglycemia

Hypoglycemia triggers counter regulatory hormones (glucagon, epinephrine, growth hormone and cortisol) to maintain blood glucose at a physiological level. Hypoglycemic patients tend to present with sympathoadrenal symptoms (tachycardia, tremors, hunger, palpitations, and anxiety) followed by neuroglycopenic symptoms (confusion, blurred vision, irritability, dizziness, seizures, and coma) (Languren et al. 2013). It is important to note that recurrent or even a single episode of hypoglycemia could induce failure of the counter regulatory hormonal process, triggering severe hypoglycemia (Cryer 2006). Therefore, all patients on insulin therapy and those who carry a high risk of hypoglycemia, including those with renal insufficiency should be closely monitored with hourly blood sugar monitoring. POC blood glucose testing should be performed on any patient presenting with neurological signs or symptoms in the perioperative period. Furthermore, close attention should be paid to preoperative insulin dosing to prevent nutrition-insulin mismatch. Under anesthesia, both sympathoadrenal and neurological symptoms could be blunted. A high index of suspicion and close monitoring are valuable tools to recognize and treat hypoglycemia in a timely manner in the perioperative setting.

An international multicenter cohort study evaluating the three domains of glycemic control (hyperglycemia, hypoglycemia, and glucose variability) in critically ill patients observed an increased risk of mortality in diabetic patients with a mean blood glucose of 80–110 mg/dl (4.4–6.1 mmol/L) when compared to a mean blood glucose of 110–140, 140–180, or even > 180 mg/dl (Krinsley et al. 2013). CPOC recommends initiation of treatment for hypoglycemia if the blood glucose level is less than 108 mg/dl (6 mmol/L) in patients with diabetes on a glucose lowering medication (CPOC 2022). Along the lines of “one size does not fit all”, it is important to note that despite these specific thresholds, there could be individual variations in the occurrence of hypoglycemia symptoms in patient’s depending on their preoperative diabetic status and glycemic control. Often patients are aware of the blood glucose levels wherein they start experiencing symptoms of hypoglycemia, which could be above the levels commonly delineated, especially if their preoperative glycemic control is suboptimal. It is imperative for the perioperative clinicians to maintain the blood glucose levels above the ascertained hypoglycemic threshold.

### Treatment of hypoglycemia

Depending on the severity of neuroglycopenic symptoms, airway protection should be ensured. Glucose supplementation in the form of glucose tablets or carbohydrate rich food is provided to patients who have mild to moderate symptoms and in whom oral intake is feasible. In patients with severe symptoms or in whom oral intake is not possible, intravenous dextrose, 25 g of 50% dextrose is given as an initial dose which could be repeated as needed. If the therapeutic effect of the bolus doses of dextrose is not sustained, an infusion may be required. Glucagon 1 mg, given either intravenous or subcutaneous is an option in patients in whom the hypoglycemia is either refractory or severe (Cryer et al. 2009). Once these therapeutic measures have been taken, a detailed evaluation into the cause of the hypoglycemia should be undertaken to prevent its recurrence (Sreedharan and Abdelmalak 2017).

### Prevention of hypoglycemia

Utilization of intensive insulin regimens and tight glycemic goals are linked to the development of hypoglycemia. More conventional insulin regimens targeting blood glucose less than 180 mg/dl, appropriate preoperative dosing of oral hypoglycemic agents and insulin, implementation of appropriate fasting regimens based on timing of surgery and close monitoring of blood glucose during the perioperative period aid in the prevention of hypoglycemia.

### Diabetic ketoacidosis (DKA)

The NaDIA harms 2020 England identifies DKA as a significant adverse event that befalls inpatients admitted with diabetes (16% of all harms reported). The goal is to reduce the occurrence of these serious events including hypoglycemia rescue, DKA, Hyperosmolar Hyperglycemic State (HHS) and Hospital acquired inpatient Diabetic Foot Ulcer (DFU) through Quality Improvement work in the NHS trust hospitals. According to the 2020 report 92.1% of the patients with DKA were emergency admissions and 21.1% of the admissions were surgical (National Diabetes Inpatient Audit 2021). A diagnosis of DKA is established when the following three criteria are met (Hallett and Levy 2016):

- Known diabetes or blood glucose over 11 mmol/L (198 mg/dl),
- Blood ketones over 3 mmol/L or urine ketones 2+ or more
- Acidosis with venous PH less than 7.3 or bicarbonate less than 15 meq/L.

Noncompliance or the presence of coexisting pathological states including infection or hypovolemia can precipitate the occurrence of DKA (Hallett and Levy 2016; Levy and Dhatariya 2017). If DKA is recognized preoperatively in a patient presenting for elective surgery, the cause for DKA should be identified and surgery should be postponed until DKA is managed appropriately and resolved. If the surgery is emergent, and the patient has DKA, the management of DKA starts preoperatively and continues as the patient proceeds to the operating room (Levy and Dhatariya 2017). Fluid resuscitation with a balanced solution and administration of insulin infusion are pivotal in the management of DKA. Current guidelines suggest the utilization of weight based Fixed Rate Intravenous Insulin Infusion (FRIII) instead of a Variable Rate Intravenous Insulin Infusion (VRIII). Close monitoring and replacement of electrolytes, especially potassium is of paramount importance in the treatment of DKA. FRIII is continued until ketosis resolves (Hallett and Levy 2016; Levy and Dhatariya 2017). An endocrinology consult would be valuable in the institution of appropriate insulin therapy after the discontinuation of the FRIII.

## Conclusion

Hyperglycemia and hypoglycemia have a profound impact on mortality and morbidity. Appropriate preoperative evaluation and optimization of patients with a diagnosis of diabetes is imperative and can positively influence perioperative outcomes. Currently, there is no evidence to support perioperative intensive insulin therapy and tight glucose control in either cardiac or non-cardiac surgical patients. A moderate glycemic target of maintaining blood glucose between 140 and 180 mg/dl (7.7–10 mmol/L) with conventional insulin therapy results in fewer episodes of hypoglycemia and improved survival.

## Data Availability

Not applicable.

## Abbreviations

ACE: American College of Endocrinology
ACCE: American College of Clinical Endocrinologists
ACP: American College of Physicians
ADA: American Diabetes Association
DPP-4: Dipeptidyl peptidase-4
HbA1C: Hemoglobin A1C (HbA1C)/Glycated Hemoglobin
ICU: Intensive care unit
IIT: Intensive insulin therapy
PACU: Post-Anesthesia Care Unit
POC: Point of care
SAMBA: Society of Ambulatory Anesthesia
SCCM: Society of Critical Care Medicine
STS: Society of Thoracic Surgeons’
TDD: Total daily dose
